# Date palm fruits (*Phoenix dactylifera* L.): nutrition, phytochemistry, and translational gaps in reported bioactivities — a critical narrative review

**DOI:** 10.3389/fpls.2026.1806447

**Published:** 2026-04-13

**Authors:** Emad M. Abdallah, Abdulrahman Mohammed Alhudhaibi, Ammar Cherif, Hammad Ahmad Jan

**Affiliations:** 1 Department of Biology, College of Science, Qassim University, Buraydah, Saudi Arabia; 2 Department of Biology, College of Science, Imam Mohammad Ibn Saud Islamic University (IMSIU), Riyadh, Saudi Arabia; 3 Department of Botany, University of Buner, Swari, Pakistan

**Keywords:** biological activity, date palm, food, natural products, nutraceuticals

## Abstract

*Phoenix dactylifera* L. (date palm) fruits are widely consumed and valued for their nutritional and phytochemical composition. This review provides a critical narrative synthesis of current evidence on the nutritional value, phytochemical diversity, and reported biological activities of date palm fruits, with attention to the strength and relevance of available evidence. The synthesis was informed by targeted searches of PubMed, Scopus, and Google Scholar, and evidence was interpreted according to translational relevance, with priority given to human data over animal, *in vitro*, and in silico evidence. Date fruits are rich in carbohydrates, dietary fiber, essential minerals, and a wide range of phytochemicals, including phenolic acids, flavonoids, carotenoids, and phytosterols. Many experimental studies report antioxidant and metabolism-related bioactivities, and some suggest possible roles in glycemic regulation and microbial inhibition. However, most reported effects are based on *in vitro* studies, while *in vivo* and human evidence remains limited. In addition, large differences in cultivar, ripening stage, processing conditions, and analytical methods make direct comparison between studies difficult. By clearly distinguishing between levels of evidence, this review highlights important limitations and research gaps in the current literature. Future studies should focus on standardized cultivar description, improved chemical profiling, realistic intake levels, and biologically meaningful outcomes. Addressing these issues will help clarify the functional significance of date palm fruits and support their evaluation within plant science and food research frameworks.

## 1 Introduction

The global rise in chronic diseases, including cardiovascular disorders, diabetes, cancer, stroke, and age-related conditions, has intensified the demand for natural and effective healthcare alternatives, alongside a broader shift in dietary patterns (Hacker, 2024). Historically, plants have served as indispensable components of human life, not only due to their ability to synthesize their own nutrients but also because of their multifaceted roles in providing food, medicine, sustenance, and shelter (Usman et al., 2014). In recent decades, growing scientific and public interest has focused on food not merely as a source of basic nutrition, but as a determinant of health and disease prevention. Nutritional inadequacies, food scarcity, and excessive dependence on a limited number of staple crops have been linked to deficiency-related illnesses, while overconsumption of modern Western diets has contributed to the increasing prevalence of metabolic syndrome, obesity, type 2 diabetes, cardiovascular disease, and certain malignancies (Martin et al., 2013). Consequently, attention has shifted toward identifying dietary components that extend beyond caloric value to confer functional and protective health benefits (Ullah and Khan, 2008). The availability and diversity of such plant-based resources are strongly shaped by climatic and ecological factors. Within this context, date palm fruits offer a particularly relevant focus because they combine longstanding nutritional importance in arid regions with substantial phytochemical diversity and emerging interest in functional food research (Vayalil, 2012).Among ancient cultivated plants, the date palm (*Phoenix dactylifera* L.) occupies a unique position, although many aspects of its early domestication and spread remain insufficiently understood. Among plant foods of longstanding nutritional and cultural importance, the date palm holds a distinctive place, particularly in arid and semi-arid regions where it has long contributed to diet, food security, and traditional health practices (Tengberg, 2012; Taleb et al., 2016). The date fruit has long been recognized as a nutritionally dense food and a potential therapeutic resource, with a history extending back thousands of years to regions considered the cradle of civilization, particularly the Middle East (Tengberg, 2012). In the arid and semi-arid regions of North Africa, the Levant, and the Arabian Peninsula, the date palm has played a central role in sustaining human populations, contributing to food security and social stability. Traditional medical systems across these regions have extensively employed date fruits for the management of digestive disorders, fever, edema, bronchitis, and wound healing, reflecting their perceived medicinal value (Taleb et al., 2016). The significance of dates is closely tied to their high nutritional content, which historically protected populations inhabiting harsh desert environments from starvation and malnutrition.

Ecologically, the date palm is well adapted to extreme climatic conditions, thriving in environments characterized by high temperatures, low rainfall, and saline or nutrient-poor soils. Its deep root system enables access to groundwater, a critical requirement for growth and fruit production in arid landscapes (Terral et al., 2012). Archaeological evidence from Mesopotamia suggests that date cultivation dates back to approximately 3000 BCE, although the precise geographical origin of the species remains uncertain due to the long history of cultivation, exchange, and diversification of date palm varieties. It is generally believed that *Phoenix dactylifera* L. originated either in ancient Mesopotamia (southern Iraq) or western India (Wrigley, 1995).

Beyond its agricultural and nutritional importance, the date palm holds profound cultural, spiritual, and economic significance in Middle Eastern societies. Its prominence is reflected in ancient Assyrian and Babylonian records, as well as in pre-Islamic and Islamic traditions, where dates were esteemed for their horticultural, nutritional, medicinal, architectural, environmental, and economic value (Jaradat, 2013). Despite extensive ethnopharmacological use and cultural reverence, a comprehensive and critically synthesized understanding of the phytochemical composition of dates and their therapeutic relevance to contemporary health challenges remains limited. Although dates are processed and consumed in diverse forms, their potential medicinal properties remain relatively underrecognized in Western and Far Eastern contexts, largely due to limited cultivation, consumption, and a scarcity of robust scientific and clinical investigations in these regions (Vayalil, 2012). Throughout this review, reported nutritional and health-related findings are interpreted according to translational relevance, with human studies considered most informative, followed by animal models, *in vitro* assays, and in silico predictions. Preclinical findings are treated as hypothesis-generating unless supported by *in vivo* or human evidence.

The current review aims to critically synthesize available evidence on the nutritional composition, phytochemical diversity, and reported biological activities of *Phoenix dactylifera* L. fruits. Emphasis is placed on distinguishing between human, animal, *in vitro*, and in silico evidence, evaluating the consistency and limitations of reported bioactivities, and identifying key methodological and biological factors that influence comparability across studies. By highlighting translational gaps and research priorities, this review seeks to clarify the extent to which preclinical findings may inform future nutritional and biomedical investigations.

## 2 Methodology

This article was prepared as a critical narrative review aimed at synthesizing current evidence on the nutritional composition, phytochemical profile, and reported biological activities of *Phoenix dactylifera* L. fruits. Literature was identified through targeted searches of PubMed, Scopus, and Google Scholar using combinations of keywords related to date palm fruits, nutrition, phytochemistry, and biological activity. One representative search string was: (“*Phoenix dactylifera*” OR “date palm” OR dates) AND (fruit OR flesh) AND (nutrition OR phytochemical OR phenolic OR flavonoid OR carotenoid OR bioactive OR antioxidant OR antidiabetic OR antimicrobial OR anticancer). Studies were selected on the basis of relevance, methodological clarity, and biological plausibility rather than exhaustive systematic screening. The search was last updated in February 2026. Overall, the narrative synthesis drew on about 100 studies and reviews. Inclusion criteria comprised peer-reviewed original research articles and reviews reporting nutritional composition, phytochemical characterization, or biological effects of date palm fruits. Human studies, animal models, *in vitro* experiments, and in silico analyses were considered when they contributed mechanistic or functional insights. Exclusion criteria included studies focusing primarily on non-fruit parts of the date palm, agricultural or industrial applications without health relevance, comparative analyses lacking specific emphasis on *Phoenix dactylifera* fruits, duplicate publications, and articles without accessible full text. Evidence was interpreted according to translational relevance, with priority given to human data, followed by animal studies, *in vitro* experiments, and in silico predictions. Reported health-related effects were discussed conservatively and framed according to the strength and consistency of the available evidence, with particular attention to variability arising from cultivar differences, ripening stage, processing, extraction methodology, and study design.

## 3 Botanical background and consumption relevance

The date fruit is among the earliest cultivated crops, with evidence of cultivation in North Africa and the Middle East extending back at least 5,000 years. Archaeological findings from Mesopotamia indicate that date palm cultivation was practiced around 3000 BCE. The exact geographical origin of the date fruit cannot be determined with certainty due to its long history of cultivation and the extensive movement and exchange of date cultivars across regions. Existing evidence suggests that early consumption of dates occurred in either the Mesopotamian region (present-day southern Iraq) or western India (Chao and Krueger, 2007).

Dates are produced by the date palm (*Phoenix dactylifera* L.), which belongs to the family Arecaceae (palm family). The Arecaceae family comprises approximately 2,600 species distributed among 181 genera and occurs predominantly in desert, tropical, and subtropical regions. Members of this family display a wide range of growth forms, including grass-like, shrubby, and large woody species, reflecting adaptation to diverse ecological conditions. Several species within the Arecaceae produce edible fruits of different structural types, including indehiscent nuts, berries, and drupes, such as those produced by date palms, oil palms, coconuts, and the true sago palm (de Souza et al., 2020; Morais et al., 2022).

**Figure 1:**
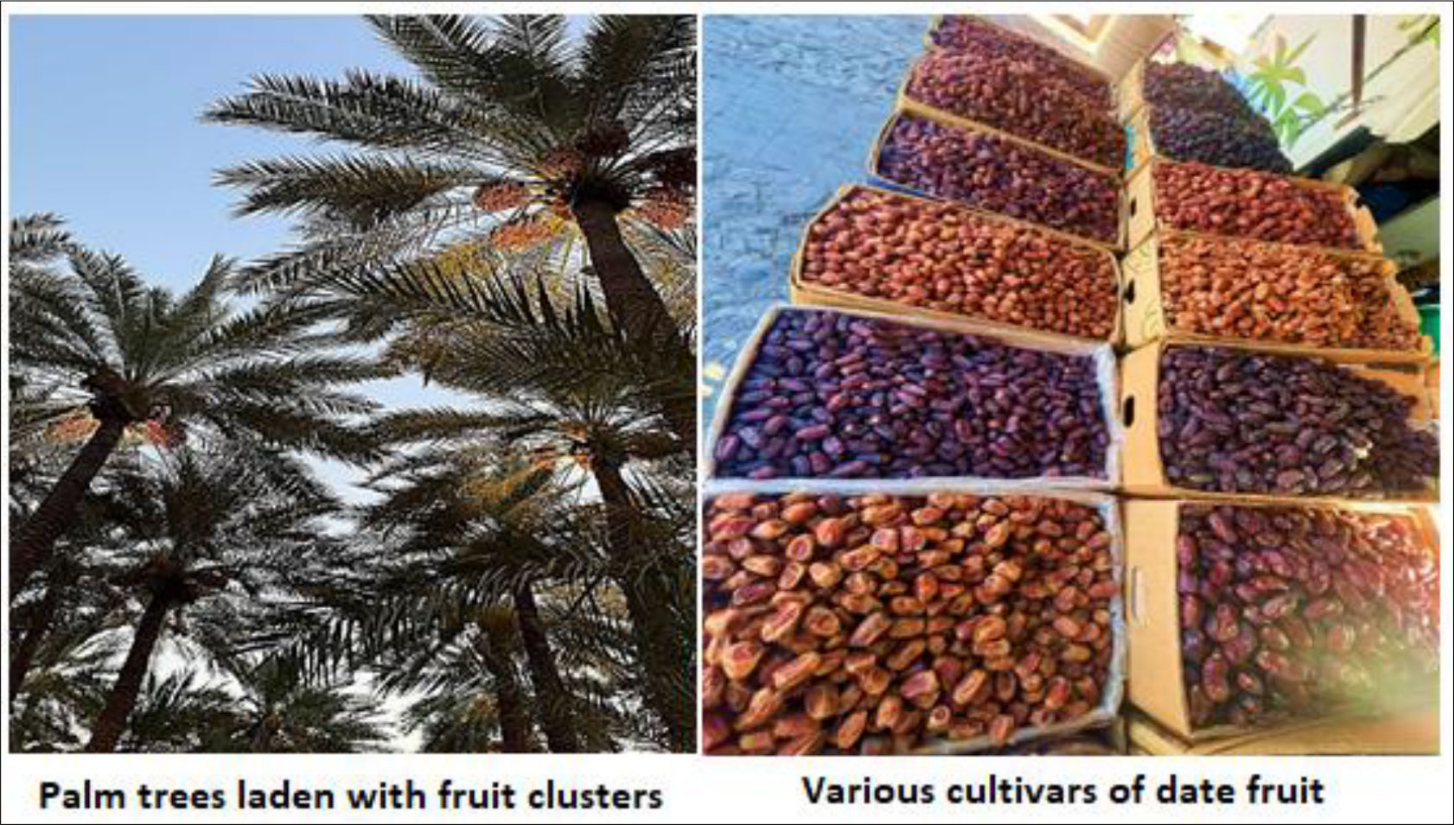
The date palm (*Phoenix dactylifera*) and its delicious dates fruits from Saudi Arabia (Photos taken by the 1^st^ author).

More than 200 date fruit varieties are cultivated worldwide, with a large proportion originating from the Middle East and North Africa (Świąder et al., 2020). Commonly cultivated varieties include Deglet Noor, Medjool, Barhi, Mazafati, Ajwa, Kholas, and Sukkary, which differ in size, texture, sweetness, and color. For example, Mazafati and Medjool are generally characterized by a softer texture, whereas Deglet Noor is comparatively firmer and exhibits a lighter flavor profile (Kuras et al., 2020; Świąder et al., 2020; Latif et al., 2024) (**Figure 1**). Over the last decade, global date palm production has increased by 18%, reaching approximately 8.53 million tons cultivated over an area of 1.11 million hectares. This increase coincides with expanded cultivation, particularly in the Middle East, and efforts aimed at improving yield and fruit quality (Alotaibi et al., 2023). In addition to direct consumption, dates are used in cooking and baking, particularly in Middle Eastern cuisine, and are also incorporated into cosmetic products and health-related applications (Nehdi et al., 2018). Their dietary relevance is also reflected in measured intake patterns in the region. In a survey of 2,177 Saudi adults, mean date consumption was approximately 100 ± 2.31 g/day, while a pilot study in Al-Ain, United Arab Emirates, reported an average intake of 8 dates/day and found that 63% of participants consumed dates daily (Al-Mssallem, 2018; Qazaq and Al Adeeb, 2010). This regional importance is also relevant to compositional research, because cultivar and ripening stage influence not only sensory traits but also the nutritional and phytochemical profiles discussed in the following sections (Ahmed et al., 1995; Abdul-Hamid et al., 2020).

## 4 Global production and nutritional significance

**Figure 2:**
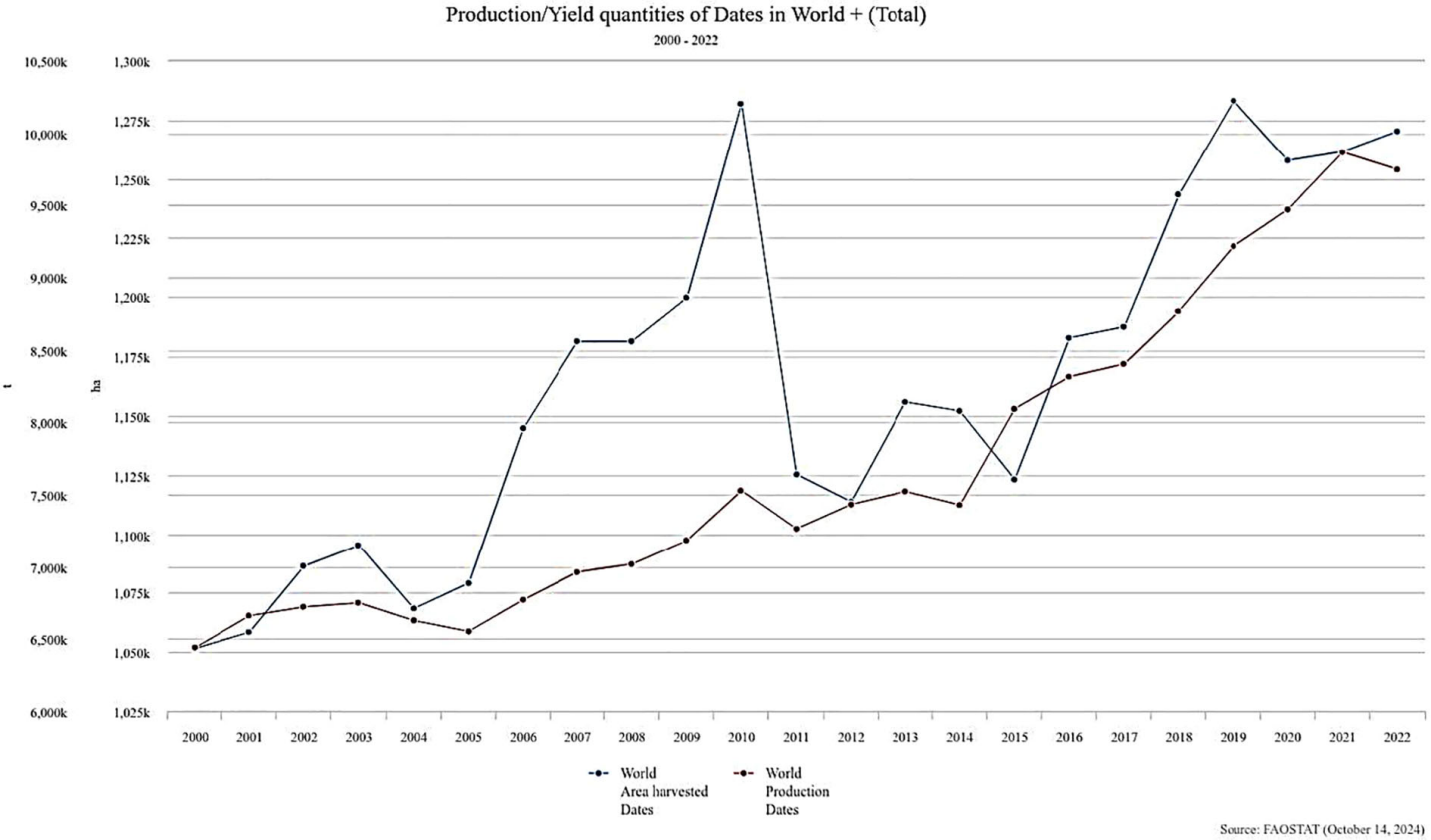
World harvested areas and Production quantities of Dates by country between 2000 – 2024 (Generated from: https://www.fao.org/faostat/en/#data/QCL/visualize Date accessed: 7/2/2026).

**Figure 3:**
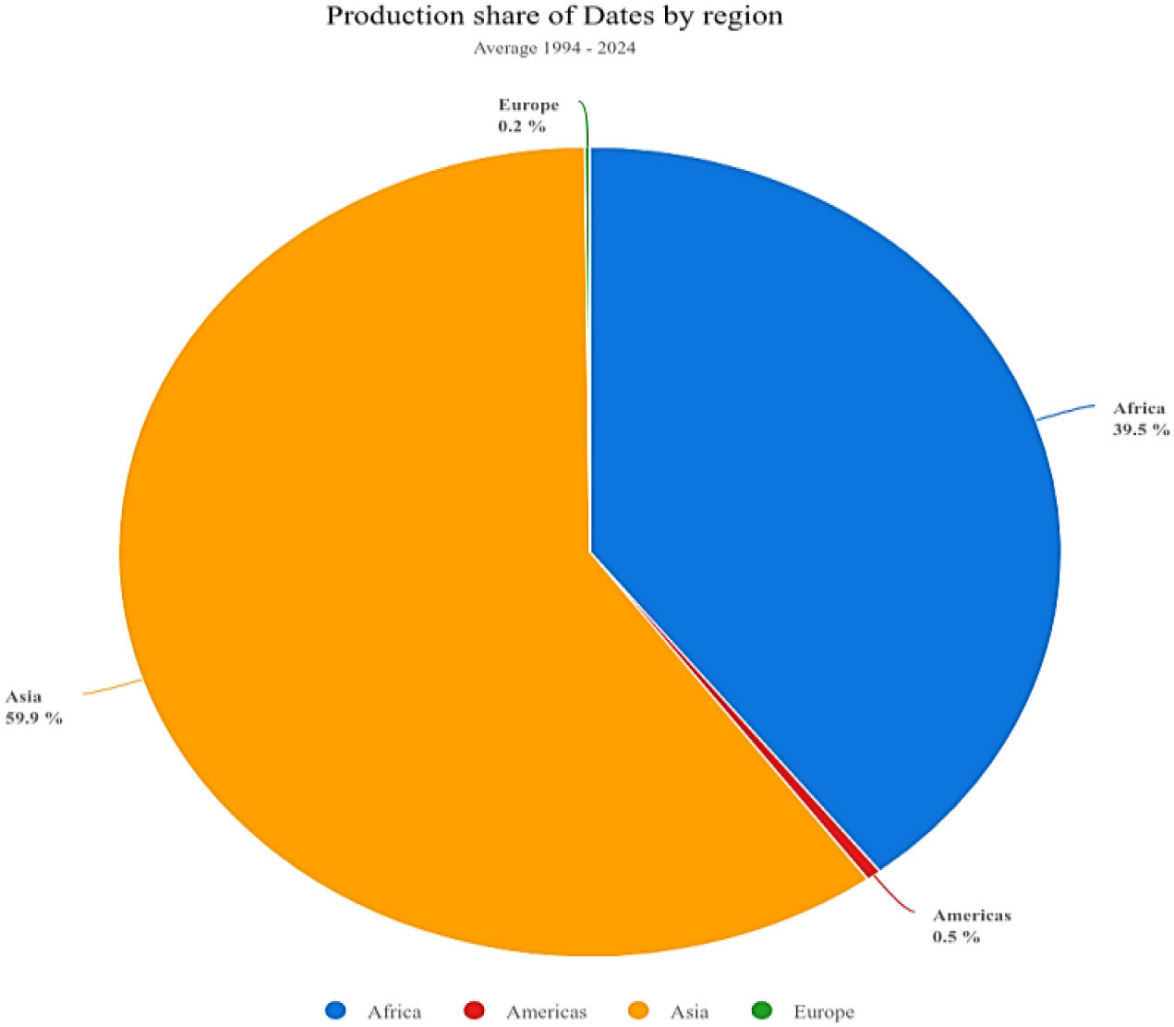
Production share of Dates by region between 2000 – 2024 (Generated from: https://www.fao.org/faostat/en/#data/QCL/visualize Date accessed: 7/2/2026).

The cultivation of dates has received considerable global attention, resulting in a year-by-year increase in production (**Figure 2**). Asia and Africa are the primary continents for date production (**Figure 3**). The domestication of the date palm coincided with the onset of agriculture. The date palm has become an important tree crop worldwide due to the application of advanced cultivation technologies and widespread human propagation, particularly through the establishment of modern plantations (Alotaibi et al., 2023). According to the Food and Agriculture Organization (FAO), from 2000 to 2020, global date production increased substantially, with Egypt, Saudi Arabia, Iran, and Iraq identified as the leading producers. Egypt, specifically, has maintained a leading role in global production, achieving annual yields exceeding 1.7 million metric tons (FAO, 2022).

## 5 Macronutrients, micronutrients, and variability

The nutritional importance of dates stems from their rich composition of carbohydrates, fatty acids, amino acids, proteins, vitamins, dietary fiber, antioxidants, salts, and minerals. Dates have also been reported to exhibit biological activities, including antimicrobial, anticancer, and antidiabetic effects. Several studies have investigated various aspects of date palm chemical composition (Al-Shahib and Marshall, 2002; Al-Farsi and Lee, 2008; El-Sohaimy and Hafez, 2010). Notably, the chemical composition of dates can vary depending on the cultivar, soil conditions, agronomic practices, and ripening stage (Al-Shahib and Marshall, 2002; Al-Farsi and Lee, 2008; Al-Kharusi et al., 2009; El-Sohaimy and Hafez, 2010).

### 5.1 Moisture dynamics and ripening-dependent changes

The moisture content of dates varies considerably depending on cultivar, ripening stage, and harvest time. During ripening, water content decreases while sugars are converted primarily to glucose and fructose, with small amounts of mannose and maltose also reported. For example, in 12 date varieties, moisture content decreases from Kimri stage (83.6 g/100 g), Khalel stage (65.9 g/100 g), Rutab stage (43 g/100 g) to Tamer stage (24.2 g/100 g) (Al-Shahib and Marshall, 2003). Hussain et al. (2020) reported that in the Hababouk stage, water content is 80–90%, decreasing to ~50% in the Khalal stage, 35–40% in the Rutab stage, and 20–25% in the Tamar stage. Similarly, Myhara et al. (1999) observed moisture contents of 21.5 and 18.1 g/100 g in the Fardh and Khasab varieties at the Tamar stage, respectively. Fresh date varieties ranged from 37.6 to 50.4 g/100 g, whereas dried varieties ranged from 7.2 to 29.5 g/100 g (Al-Farsi and Lee, 2008). Aljaloud et al. (2020) reported moisture contents in dried dates from Deglet Noor, Dabbas, Medjoul, Ajwa, and Barhi as 13.5, 19.5, 21.32, 22.8, and 29.5 g/100 g, respectively. Overall, the Tamer stage appears optimal for harvest, and Deglet Noor shows superior quality due to its lower moisture content. Variations in moisture across varieties and regions largely reflect differences in harvest timing and post-harvest treatments (Aidoo et al., 1996).

Human and post-harvest studies consistently show a decrease in water content with ripening, affecting texture, storage, and quality. These patterns are reproducible across cultivars and regions. However, variability in reported values across studies highlights the influence of local agronomic practices, ripening definitions, and measurement methods, limiting direct comparability and generalization.

### 5.2 Carbohydrate and sugar composition

**Figure 4:**
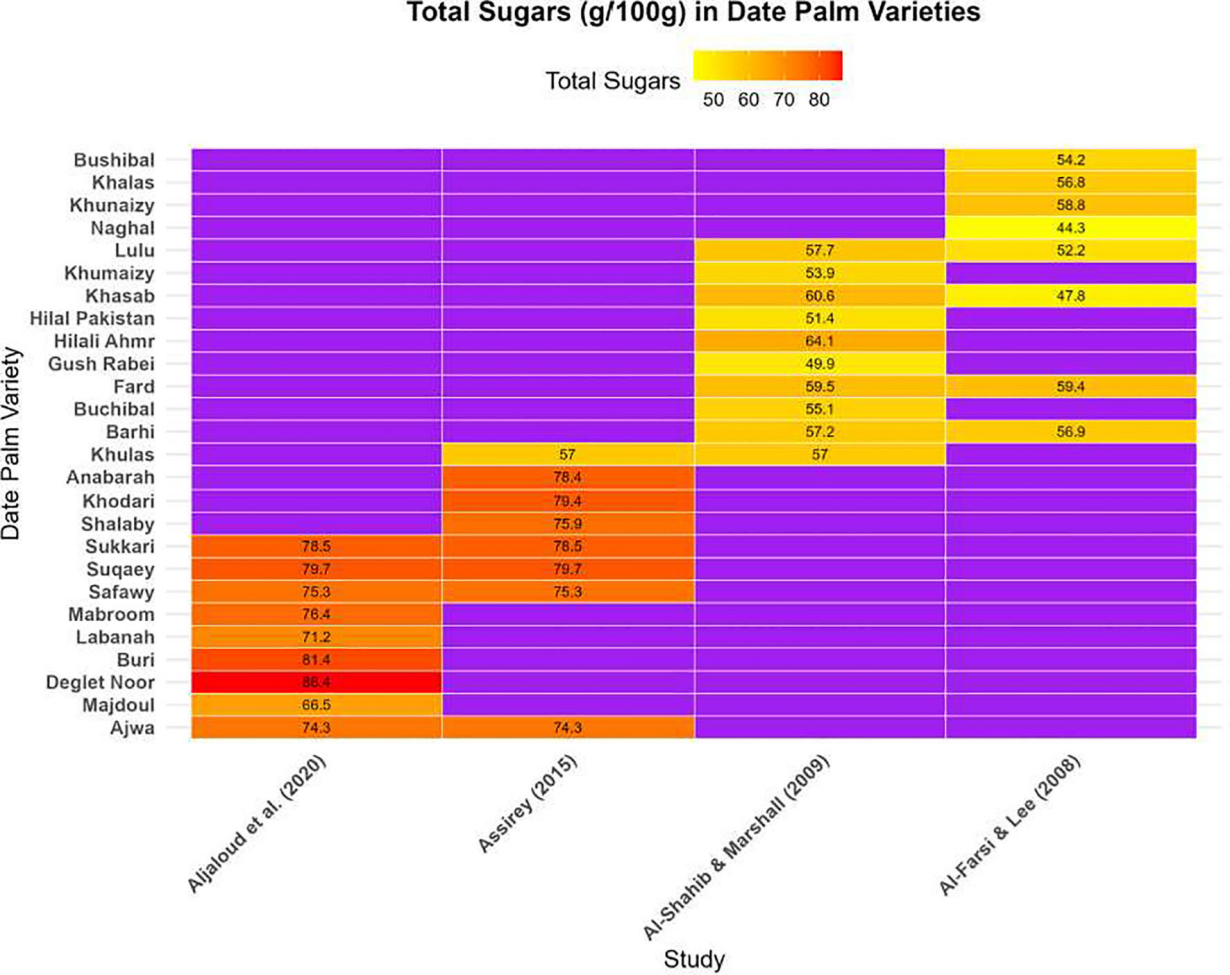
Total sugar composition (g/100 g dw) in different palm varieties at the mature stage (Created by the author from **Supplementary Table S1**).

**Figure 5:**
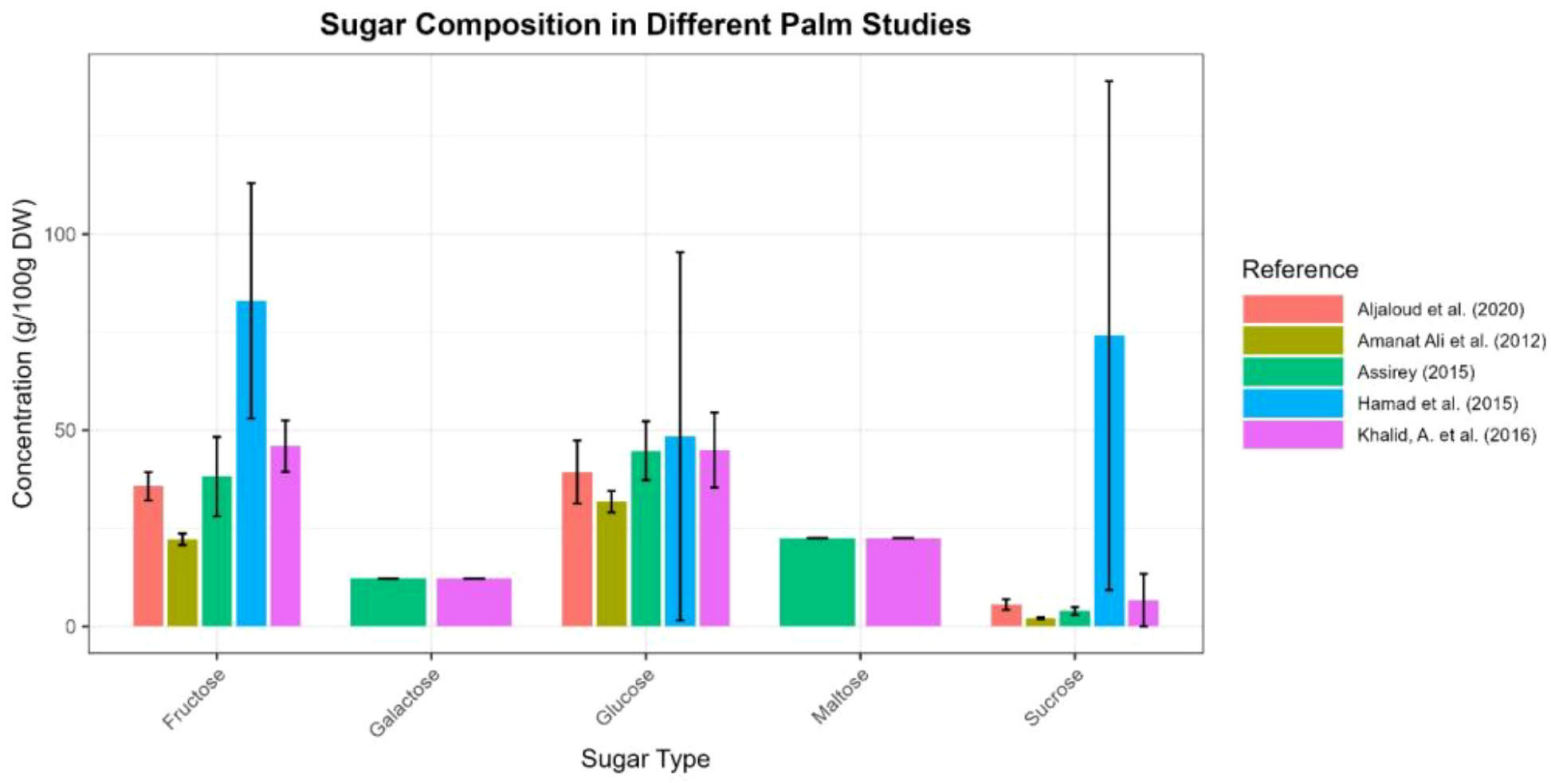
Sugar composition of different date fruit varieties according to many authors (Created by the author from **Supplementary Table S2**).

Date fruit is a high-nutritional-value food that is rich in carbohydrates and other compounds. Dates contain a high concentration of sugars, considered the main component, which can reach up to 70–80% (**Figure 4**; **Supplementary Table 1** supplementary file). These carbohydrates consist mainly of reducing sugars, including glucose, fructose, mannose, and maltose, as well as non-reducing sugars and small amounts of polysaccharides, such as cellulose and starch. Sucrose is also present in minor amounts (Al-Farsi and Lee, 2008). The total carbohydrate content depends on the ripening stage and the date variety. **Figure 4** presents the total sugar composition (g/100 g dw) in different date palm varieties at the mature stage, compiled from multiple studies. In 12 varieties of dates at different ripening stages, total sugar content ranged from 6.2 to 50.8 g/100 g (Ahmed et al., 1995). Other studies reported sugar contents ranging from 47.8 to 59.4 g/100 g in 10 date palm varieties (Al-Farsi and Lee, 2008), while Assirey (2015) observed values between 71.2 and 81.4 g/100 g in another set of 10 date varieties. Dried date fruits contain between 66.47 and 86.42 g/100 g in different varieties (Aljaloud et al., 2020). The observed variation in total sugars is largely reflected by differences in the concentration of individual sugars (**Figure 5**; **Supplementary Table 2** supplementary file). Multiple studies consistently demonstrate that sugars constitute the dominant macronutrient in dates, with content varying by variety, ripening stage, and drying process. Reducing sugars (glucose, fructose) are the major components across all varieties, contributing significantly to energy value and sweetness. Despite general agreement on the high sugar content of dates, reported values vary widely across studies, likely due to differences in cultivar, ripening definitions, analytical methods, and post-harvest processing. These variations limit direct comparisons and highlight the need for standardized reporting (Ahmed et al., 1995; Al-Farsi and Lee, 2008; Assirey, 2015).

### 5.3 Protein content and amino acid profile

**Figure 6:**
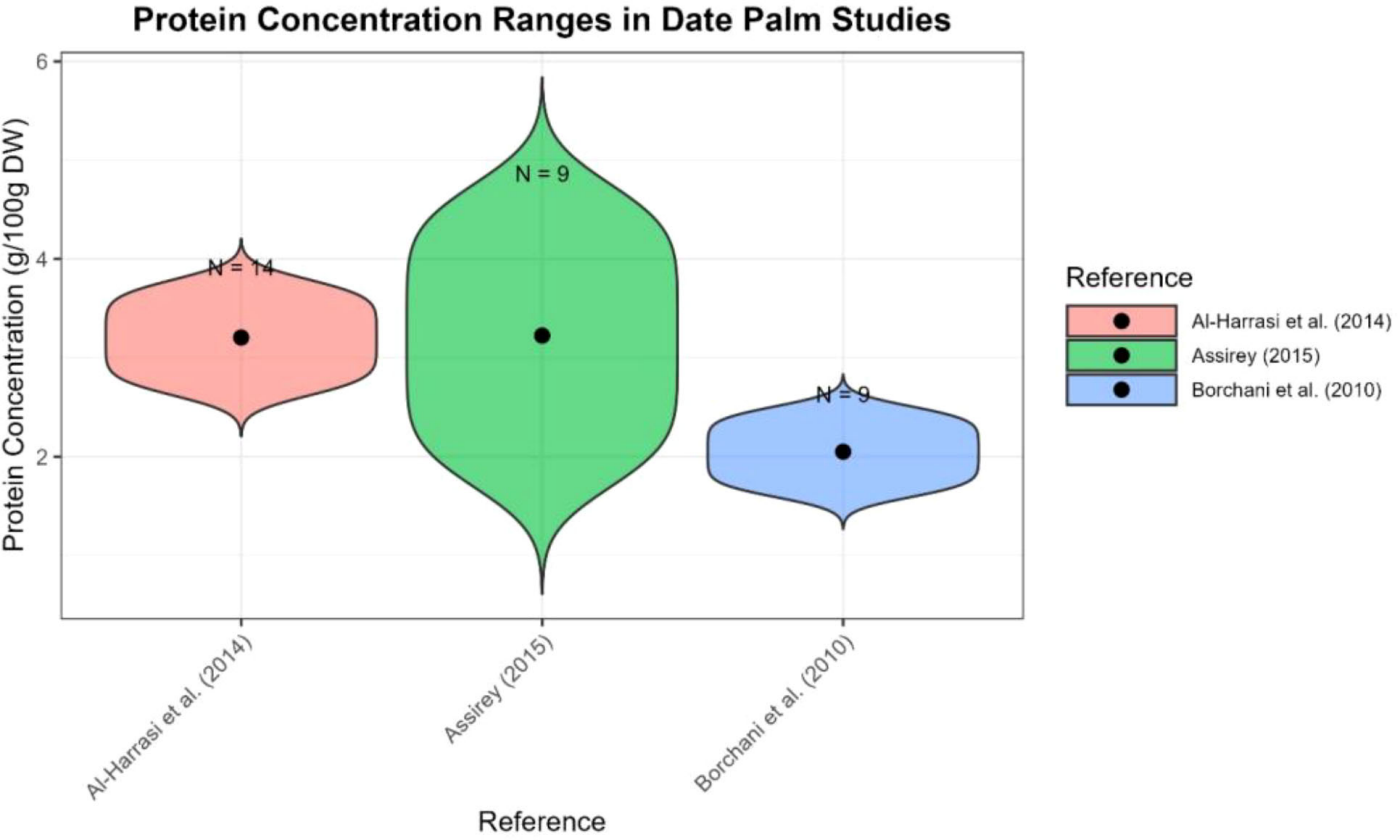
Protein content in different cultivar of date palm fruit (Created by the author from **Supplementary Table S3**).

Different date varieties are good sources of proteins and provide a range of amino acids. Dates contain a higher percentage of protein than many other fruits. However, protein concentrations differ between varieties due to differences in cultivation, drying conditions, and analytical methods used for determination. The average protein content of fresh and dried dates is 1.50 and 2.14 g/100 g, respectively. Sablani et al. (2008) reported a high percentage up to 7%, compared to Al-Shahib and Marshall (2003) (2.3–5.6%). Mekki et al. (1998) reported that the protein content of the pulp ranged between 1.7% and 2.95% on a fresh weight basis, whereas the date seeds on average contained 5.22%. Although the absolute protein amount is relatively low, dates contain many essential amino acids, and their amino acid profile is favorable for human nutritional needs. Borchani et al. (2010) reported crude protein content of 1.6–2.5 g/100 g dw, compared to ranges published elsewhere of 1.72–4.73 g/100 g dw (Assirey, 2015). Protein and amino acid composition obtained from different cultivars of date palm fruit are shown in **Figure 6**; **Supplementary Table 3** supplementary file.

**Figure 7:**
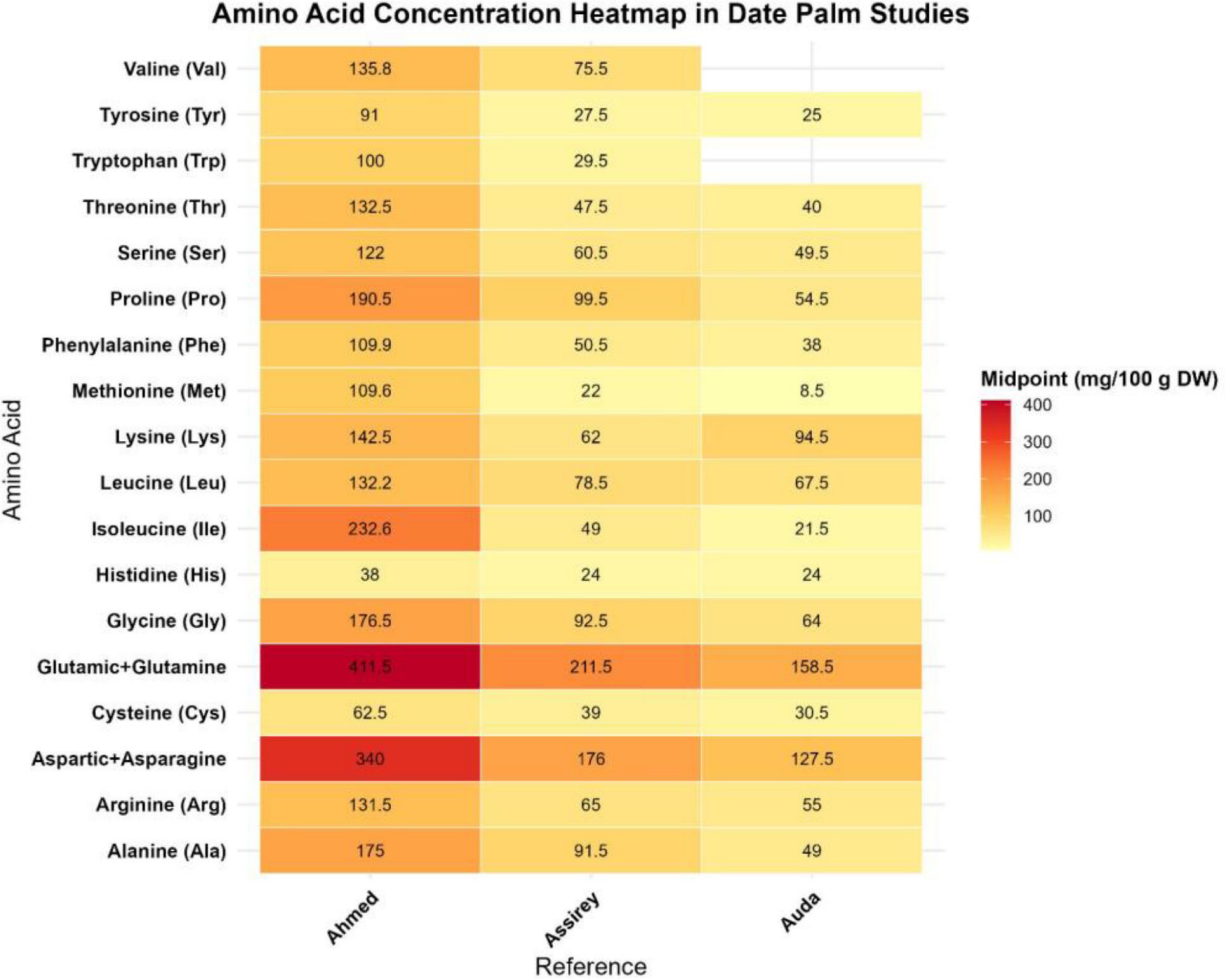
Amino acid concentration of date palm fruit reported from different studies (Created by the author from **Supplementary Table S4**).

The protein content of dates plays a critical role in non-oxidative browning and the precipitation of tannins during ripening (Mekki et al., 1998). More than twenty amino acids have been identified in date proteins. Ishurd et al. (2004) reported a reduction in amino acid contents through maturation stages. Many studies have found high levels of glutamic acid (40–631 mg/100 g dw). Other amino acids in descending concentration include alpha-amino butyric acid, isoleucine, asparagine, proline, glycine, and alanine (Al-Shahib and Marshall, 2003) (**Figure 7**; **Supplementary Table 4** supplementary file). Assirey (2015) also reported the highest contents of the same amino acids. According to these studies, the range of glutamic acid and glutamine is 158–265 mg/100 g dw and 100–217 mg/100 g dw, respectively, while methionine (Met) consistently showed the lowest levels (4–27 mg/100 g dw).

Studies consistently demonstrate that dates are moderate sources of protein, with concentrations varying by variety, ripening stage, and post-harvest processing. Essential amino acids are present in favorable proportions for human nutrition, and glutamic acid is generally the most abundant. Although protein is present, its total contribution to daily dietary protein is limited. Wide variability exists across studies due to cultivar differences, analytical methods, and drying processes, which complicates direct comparisons. Data on amino acid changes during ripening are limited and require standardized reporting (Mekki et al., 1998; Al-Shahib and Marshall, 2002, Al-Shahib and Marshall, 2003; Ishurd et al., 2004; Sablani et al., 2008; Borchani et al., 2010; Assirey, 2015).

### 5.4 Lipid and fatty acid composition

**Figure 8:**
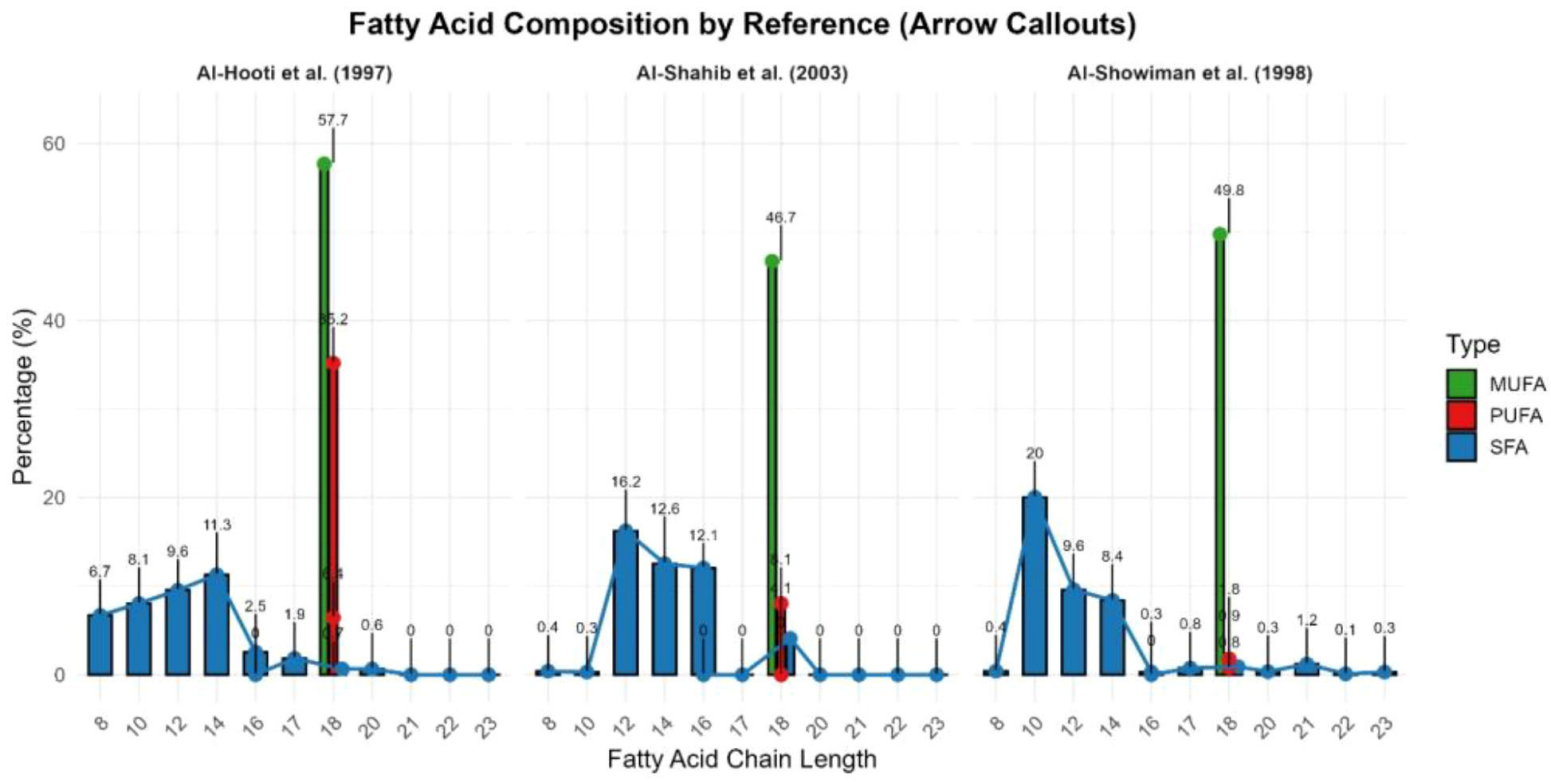
Fatty acid composition of date seeds provided from some studies(Created by the author from **Supplementary Table S5**).

The flesh of dates contains 0.2–0.5% oil, whereas the seed contains 7.7–9.7% oil. The weight of the seed represents 5.6–14.2% of the whole fruit (Al-Shahib and Marshall, 2003). Fatty acids occur in both flesh and seed, comprising saturated (SFA) and unsaturated fatty acids (UFA), with seeds containing 15 types of fatty acids. The major SFA in date seeds include lauric acid (24.7 g/100 g fat), myristic acid (14.5 g/100 g fat), and palmitic acid (13 g/100 g fat). The major UFA is linoleic acid (12.8 g/100 g), while oleic acid content ranges from 11.6 to 58.8%, suggesting that date seeds could serve as a potential source of oleic acid (**Figure 8**; **Supplementary Table 5** supplementary file). Data consistently show that date seeds are rich in both saturated and unsaturated fatty acids, particularly oleic acid, making them nutritionally and industrially valuable. The flesh contains only minor amounts of fatty acids, primarily as C18:1 and C18:2, reflecting low lipid content compared to the seed. Fatty acid content varies widely among date varieties, maturation stages, and between flesh and seed. Studies are limited on the functional impacts of date lipids on human health, and most data are based on chemical composition rather than clinical outcomes.

### 5.5 Vitamin profile and processing effects on retention

Fresh dates contain higher concentrations of vitamins compared to dry dates, as some vitamins are lost during the drying process. Dry dates contain at least six vitamins. They can be considered a moderate source of riboflavin (vitamin B2), pyridoxine, niacin (nicotinic acid), and folic acid. Thiamin (vitamin B1), ascorbic acid (vitamin C), and vitamin A are present in relatively low concentrations. Studies consistently show that vitamin levels decline during drying, with B-group vitamins (B2, B6, niacin, folic acid) being the most abundant in dried fruit, while vitamins A and C remain low (Al-Shahib and Marshall, 2003). This supports the nutritional value of fresh dates for vitamin intake. Vitamin composition varies with date variety, ripening stage, and drying method. Data on bioavailability and the effect of processing on vitamin retention are limited, and few studies provide clinical evidence on the impact of date-derived vitamins on human health.

### 5.6 Mineral composition, nutritional value, and safety

**Figure 9:**
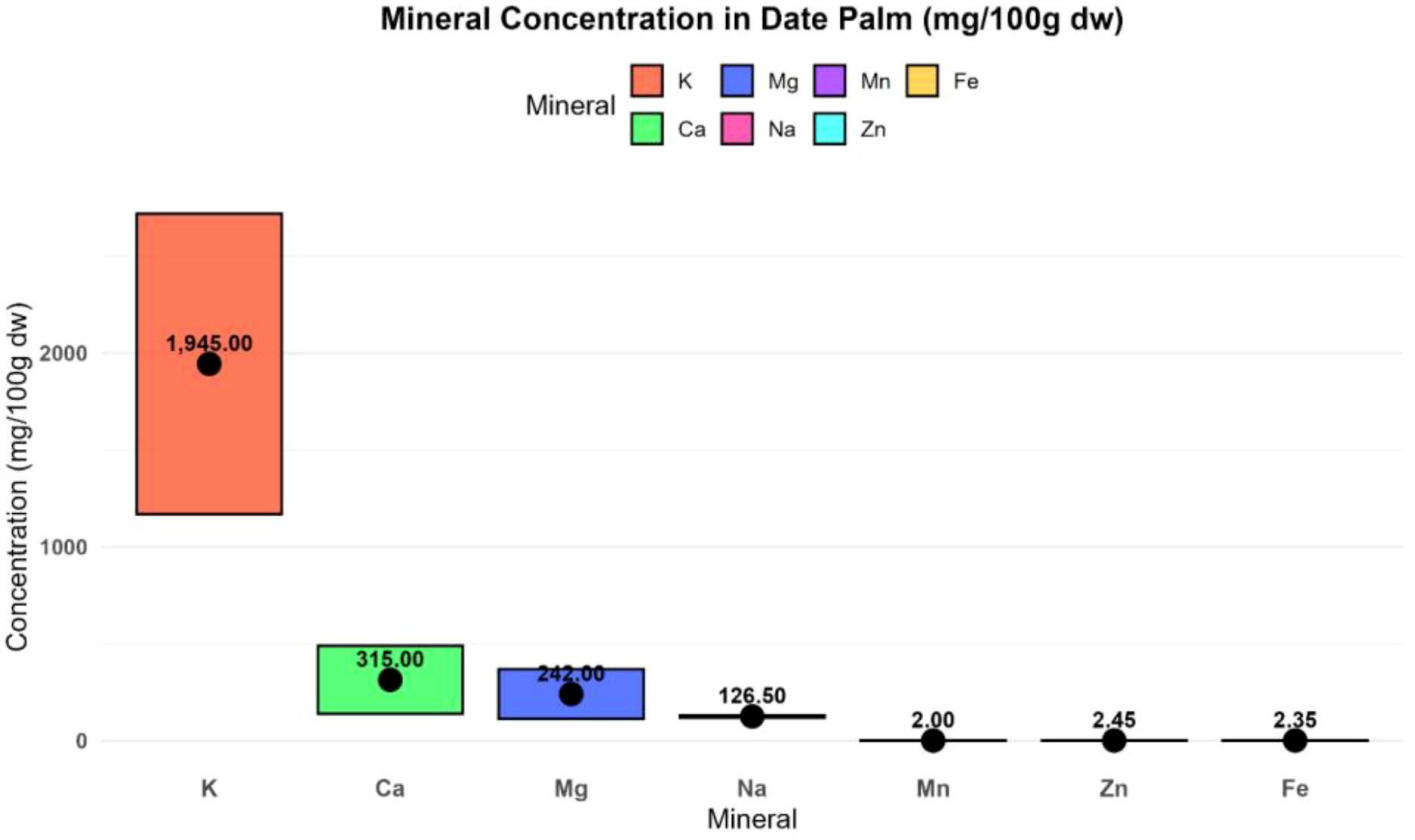
Macro and microelement concentrations of different varieties of dates at different ripening stages (10^-2^g/kg^-1^)(Created by the author from **Supplementary Table S6**).

Dates are a very good source of many minerals (**Figure 9**), with concentrations approaching the average nutrient intake requirements. There are at least 17 minerals in dates, with concentrations in the flesh varying from 0.1 to 916 mg/100 g dw and in seeds from 0.001 to 538.5 mg/100 g dw. Potassium was found at the highest concentration in both flesh and seeds, whereas cobalt and cadmium were present at the lowest levels in flesh and seeds, respectively (Al-Shahib and Marshall, 2003). The following minerals are found in varying concentrations in flesh and seeds: boron, calcium, copper, iron, magnesium, manganese, potassium, phosphorous, sodium, and zinc (**Supplementary Tables 6**, **S7**, supplementary file). Additionally, seeds contain aluminum, cadmium, and lead in various proportions, while cobalt, fluorine, and selenium are present only in the flesh (Al-Shahib and Marshall, 2003).

Some minerals provide specific health benefits. Fluorine (0.1–0.2 mg/100 g dw) protects teeth against decay, and selenium (0.1–0.3 mg/100 g dw) supports immune function and may have cancer-preventive effects (Al-Shahib and Marshall, 2003). Potassium contributes to cardiovascular health by regulating heart rate and blood pressure and is essential for fluid and nervous system balance (Haddy et al., 2006). Magnesium acts as a cofactor in multiple biochemical reactions (Pamnani et al., 2003).

**Figure 10:**
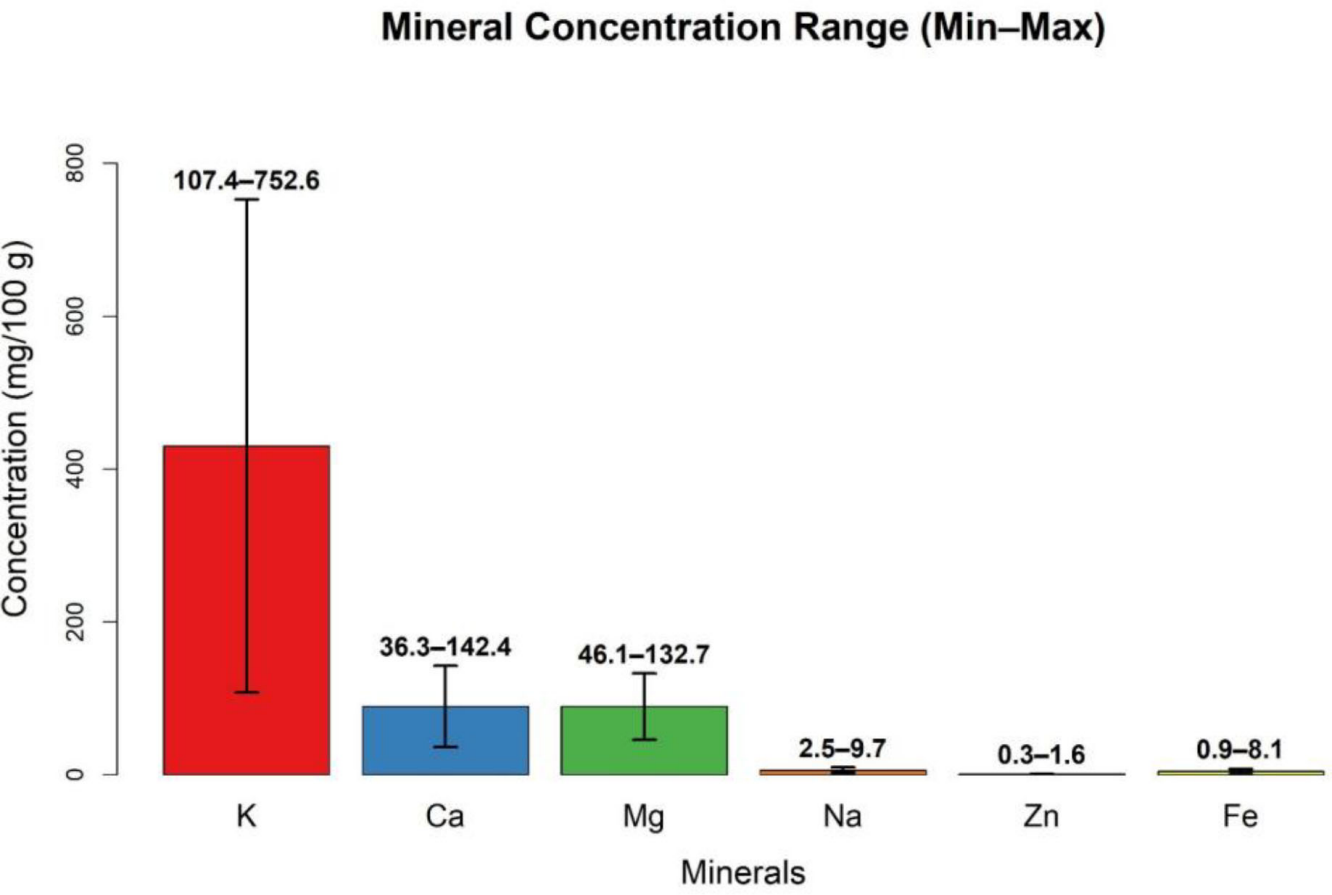
Macro and microelement concentrations of different varieties of dates at different ripening stages (mg/100g dw) (Created by the author from **Supplementary Table S7**).

Studies showed that mineral concentrations vary across different date varieties and decline progressively with ripening stages (Rastegar et al., 2012), as presented in **Figures 9**, **10** (**Supplementary Tables 6**, **7** **Supplementary File**). Multiple studies confirm that dates are rich in both macro- and micro-minerals, with concentrations influenced by variety, ripening stage, and plant part (flesh *vs* seed). Potassium, phosphorous, and iron consistently show the highest concentrations and are nutritionally significant compared to other common fruits. Mineral content is highly variable depending on cultivar, ripening stage, and post-harvest treatment. Limited clinical evidence exists on the bioavailability and health impact of date-derived minerals in humans (Rastegar et al., 2012). Some trace minerals (aluminum, cadmium, lead) may pose potential toxicity risks if consumed in high quantities.

## 6 Phytochemical diversity and variability

**Figure 11:**
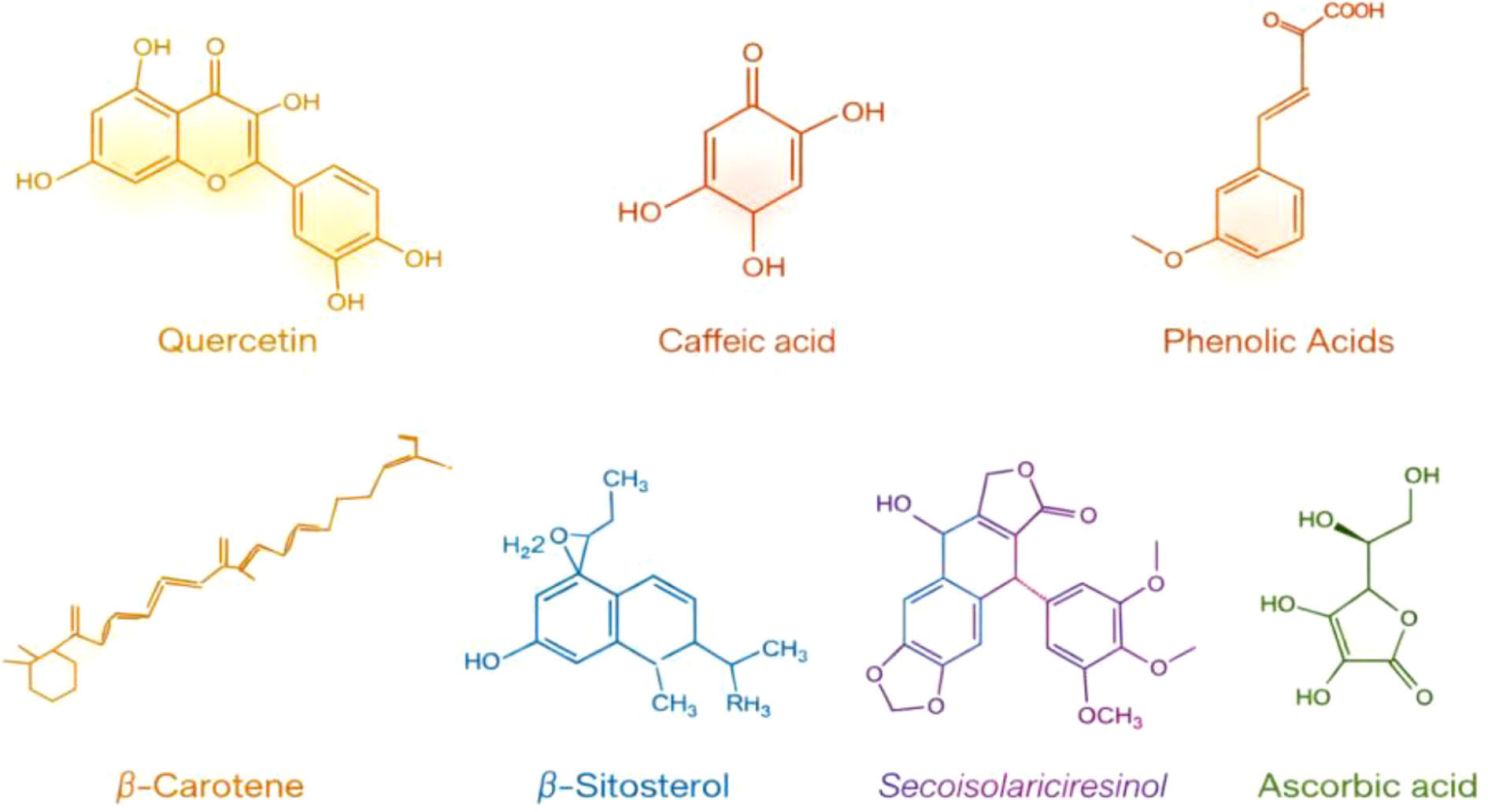
Chemical structures of some predominant bioactive compounds present in dates (Martıń-Sańchez et al., 2014; Ho, 1992; Boudries et al., 2007; Al-Farsi and Lee, 2008; Biglari et al., 2008).

The fruit of dates is rich in phytochemicals (**Figure 11**). Date fruits contain various classes of bioactive compounds, including carotenoids, polyphenols (particularly phenolic acids), isoflavones, lignans, flavonoids, tannins, and sterols. The quantity and composition of these phytochemicals can vary depending on factors such as cultivar, stage of ripeness, storage conditions, postharvest processing, degree of hydration, analytical methods, and geographical origin (Jain, 2013). For example, in Tunisia, the Korkobbi variety demonstrated the highest phenolic content (54.66 mg/100 g fresh weight), while the Mermella variety exhibited the lowest (5.73 mg/100 g fresh weight) (Chaira et al., 2009). Mansouri et al. (2005) analyzed seven Algerian date varieties and reported p-coumaric, sinapic, and ferulic acids, as well as cinnamic acid derivatives and three isomers of 5-O-caffeoyl shikimic acid. Kikuchi and Miki (1978) identified sterols including β-sitosterol, cholesterol, campesterol, isofucosterol, and stigmasterol (Kikuchi and Miki, 1978). Carotenoids such as β-carotene, lutein, and neoxanthin were reported in date fruits (Di Mascio et al., 1991), though sun-drying significantly reduces total carotenoid content in Omani varieties (Fard, Khasab, Khalas) (Al-Farsi et al., 2005). Flavonoids, including catechin and rutin, were present in significant quantities across 13 Saudi date varieties (Idowu et al., 2020).

Dates are rich in polyphenols, carotenoids, isoflavones, flavonoids, lignans, phytosterols, and tannins, all contributing to strong antioxidant potential (Martín-Sánchez et al., 2014). Major carotenoids include beta-carotene, lycopene, lutein, zeaxanthin, and neoxanthin, whereas major phytosterols are campesterol, β-sitosterol, stigmasterol, and isofucosterol, particularly at the Tamer stage (Ho, 1992; Boudries et al., 2007; Al-Farsi and Lee, 2008; Biglari et al., 2008). Phytoestrogens such as daidzein, coumestrol, genistein, glycitein, matairesinol, pinoresinol, lariciresinol, and secoisolariciresinol are also present (Thompson et al., 2006).

Phenolic acids in dates include benzoic acid derivatives (vanillic, gallic, p-hydroxybenzoic, syringic, and protocatechuic acids) and cinnamic acid derivatives (p-coumaric, o-coumaric, ferulic, and caffeic acids) (Al-Farsi et al., 2005). Flavonoids, as major polyphenolic secondary metabolites, exhibit strong antioxidant and anti-inflammatory activities (Moss and Ramji, 2016). More than 13 flavonoid glycosides (luteolin, apigenin, quercetin) and 19 isomeric flavonoids have been reported (Hong et al., 2006). The concentration of most phytochemicals declines with advancing fruit maturity, although maturity stage influences antioxidant activity.

Reported phytochemical profiles are also influenced by extraction solvent and method. To improve cross-study comparability, future work should report extraction conditions more clearly and consistently, including solvent composition, solid-to-liquid ratio, extraction time, temperature, and any pre-analytical concentration or drying steps. Better methodological standardization would help separate genuine cultivar- and maturity-related differences from variation introduced by extraction and analytical procedures. For instance, aqueous extracts of Ajwa dates show strong antioxidant properties due to hydrophilic compound enrichment (Al-Farsi et al., 2005; Saleh et al., 2011; Salehi et al., 2019). Mrabet et al. (2016) identified p-coumaric acid, gallic acid, protocatechuic acid, tyrosol, vanillic acid, and syringic acid in different varieties, while Daoud et al. (2019) reported gallic, vanillic, caffeic acids, and coumarin. Mansouri et al. (2005) additionally found caffeic acid derivatives, caffeoylshikimic acid, hydrocaffeic acid, p-coumaric acid, cerulic acid, cinnamic acid, 5-o-caffeoylshikimic acid isomers, ferulic acid, sinapic acid derivatives, gallic acid derivatives, and ginnamic acid in five date varieties. Flavonoid content, expressed as milligrams of quercetin equivalents (QEQ) per 100 g fresh weight (FW) and estimated from a quercetin standard calibration curve, ranged from 6.41 to 54.4 mg QEQ/100 g FW, with the highest levels reported in Ourrous and Deglet Nour, and lowest in Beid Lahmam and Outkbala varieties ( (Louaileche et al., 2015).). Moreover, quercetin, luteolin, apigenin, isoquercetrin, and rutin were reported in 11 varieties (Louaileche et al., 2015).

Evidence from *in vitro* and *in vivo* studies indicates that date fruits contain significant polyphenolic compounds and exhibit antioxidant activity, suggesting potential benefits in nutraceutical and functional food applications. These phytochemicals may contribute to reducing oxidative stress and potentially lowering cancer incidence and mortality rates (Shahidi and Ho, 2005). Human, animal, and *in vitro* studies consistently demonstrate that date fruits are rich in bioactive phytochemicals, particularly polyphenols, flavonoids, carotenoids, and sterols, with strong antioxidant capacity. The composition and concentration of these compounds are highly influenced by variety, maturity stage, extraction method, and processing. Most studies are *in vitro* or animal-based, with limited human clinical evidence for direct health outcomes. Phytochemical concentrations vary substantially with ripening, postharvest processing, and extraction method. Effects on disease prevention remain largely hypothetical and require rigorous clinical validation.

## 7 Biological activities: evidence and relevance

**Figure 12:**
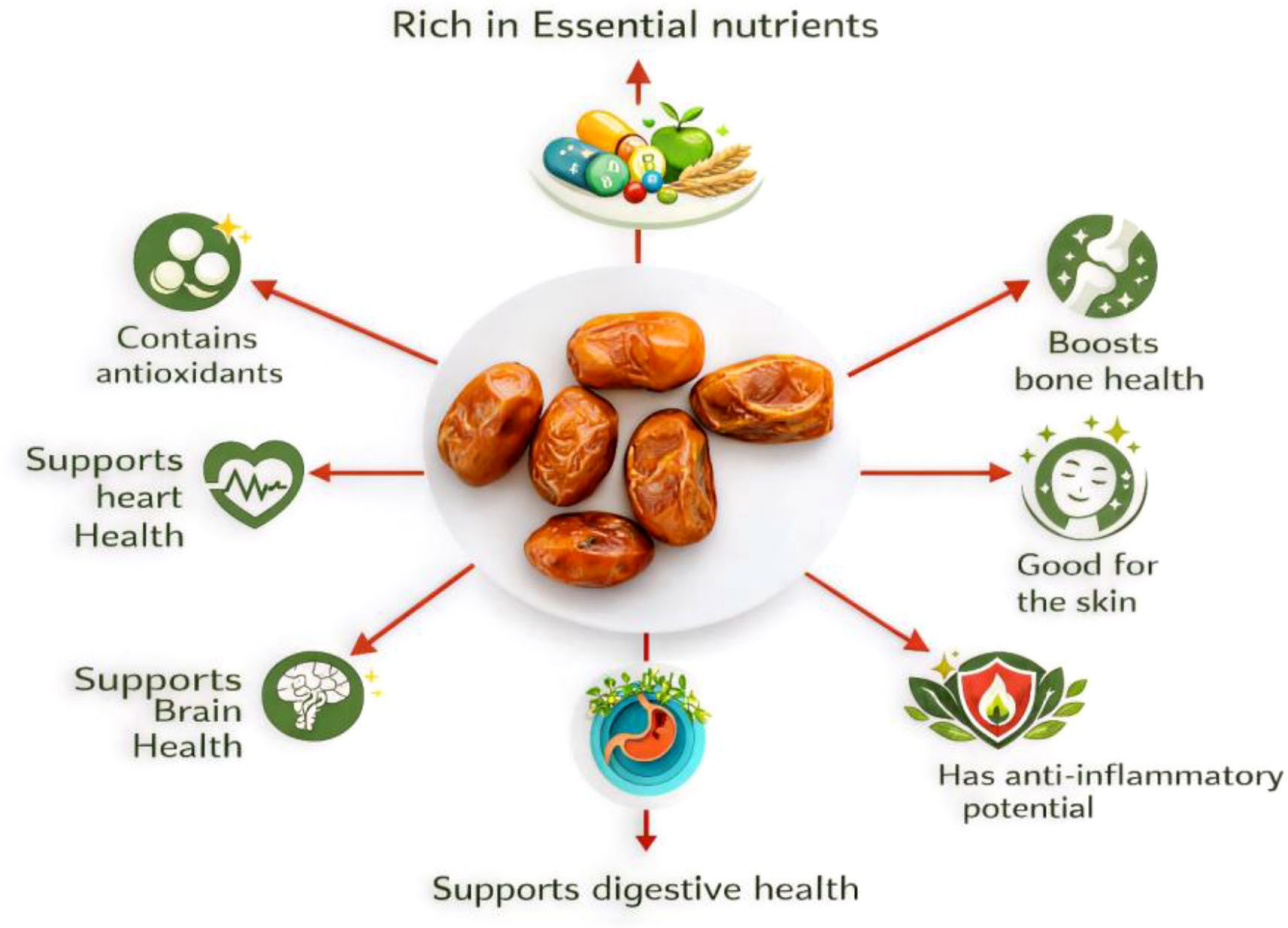
Some health benefits of dates.

dates can be considered a functional food based on their nutritional value and emerging bioactive potential (**Figure 12**). Functional foods are natural or processed foods that incorporate biologically active compounds, either identified or unidentified. When consumed in specified, effective, and safe amounts, these foods offer clinically proven and documented health benefits, aiding in the prevention, management, or prevention of chronic diseases (Martirosyan and Singh, 2015). Nutraceuticals are bioactive compounds extracted from either plant-based or animal-derived foods. They have the potential to prevent or manage various chronic diseases and are available in pharmaceutical formulations (Al-Okbi, 2022).

Research has demonstrated that dates exhibit a variety of beneficial properties, including free radical scavenging, antioxidant, anti-inflammatory, antimutagenic, gastroprotective, hepatoprotective, nephroprotective, anticancer, antiulcer, immunostimulant, and antimicrobial effects (Dayang et al., 2014).

### 7.1 Antioxidant activity: evidence and limitations

Date fruits are a rich source of antioxidants; dates possess significant antioxidant potential due to their ability to scavenge free radicals. These natural antioxidants offer numerous health benefits, including cancer prevention, protection against microbial infections and chronic inflammation, reduced risk of cardiovascular diseases, and anti-mutagenic effects (Younas et al., 2020). Most of the antioxidants observed in dates have been reported to be hydrophilic or water-soluble (Al-Farsi et al., 2005). The variability in the data about the total antioxidant content of dates could be attributed to different extraction techniques and analytical methods used for their estimation. Higher antioxidant activity has been reported in fresh dates as compared to dried dates, which may be due to the decomposition of natural antioxidants during the drying process.

Apart from their nutritional value, dates are rich in phenolic compounds having antioxidant activity. Some studies from different countries, such as Algeria, Kuwait, Iran, Bahrain, Oman, and the United States, have reported the antioxidant activity of date fruits due to their high content of active phenolic acids (Vayalil, 2002; Vinson et al., 2005; Allaith, 2008; Elleuch et al., 2008). Ou et al. (2001) reported a higher antioxidant capacity of date fruits concerning free oxygen radicals in comparison to other fruits. Additionally, dates showed the second-highest antioxidant property amongst 28 other fruits (Guo et al., 2003).

The antioxidant potential of dates is well-supported *in vitro* and in animal models, largely attributed to phenolic acids, flavonoids, and carotenoids. Comparative studies demonstrate that fresh dates generally exhibit higher antioxidant activity than dried fruits, suggesting that postharvest processing affects bioactive compound retention. Human clinical evidence for antioxidant-related health outcomes is limited. Differences in variety, ripening stage, extraction method, and analytical protocol result in high variability of reported antioxidant content, making direct comparisons challenging. Therefore, while laboratory studies indicate strong antioxidant potential, translation into measurable clinical benefits requires further well-designed human trials.

### 7.2 Antidiabetic bioactivities: human and preclinical evidence

Despite their high natural sugar content, dates have shown potential antidiabetic properties due to their rich composition of bioactive compounds. Dates contain dietary fiber, antioxidants like flavonoids and phenolic acids, and minerals, which can aid in glycemic control. These compounds may help in slowing down the absorption of glucose and improving insulin sensitivity (Barakat and Alfheeaid, 2023). A study evaluating the glycemic indices (GI) of five date varieties in both healthy and diabetic subjects revealed that all five types exhibited low GI values. The results indicated that the consumption of these dates by individuals with diabetes did not cause significant increases in postprandial blood glucose levels, suggesting that dates can be consumed without a substantial impact on glucose control in diabetic patients (Alkaabi et al., 2011).

The antidiabetic properties of dates are thought to be attributed to their ability to enhance glucose uptake, stimulate cellular glycogen synthesis, and protect pancreatic cells from damage, such as that induced by dexamethasone injections. These mechanisms collectively support improved glucose regulation and insulin sensitivity in diabetic individuals (Abdullah and Al-bokai, 2022). Moreover, aqueous date extract prevented diabetic aggravation and enhanced pathological indicators of diabetic neuropathy in diabetic rats (Zangiabadi et al., 2011). This effect may be partially related to the phenolic compounds present in dates, which inhibit α-glucosidase, thus influencing glucose absorption in the small intestines and kidneys (Khalid et al., 2017). Date-derived glycosides appear to increase insulin excretion and stimulate glycogen synthase, maintaining blood-glucose homeostasis (Singh et al., 2012).

From a translational perspective, however, a low glycemic index should not be interpreted as evidence of a therapeutic antidiabetic effect. Most mechanistic claims remain derived from enzyme-based assays or animal models, whereas controlled human intervention studies are scarce. In addition, glycemic responses are likely to vary according to cultivar, ripening stage, serving size, food matrix, and the metabolic status of the consumer (Sebastian et al., 2023).

The antidiabetic potential of dates is supported by human GI studies and multiple *in vivo* animal models. The bioactive compounds, especially phenolics, flavonoids, glycosides, and dietary fiber, contribute to improved glucose regulation, insulin sensitivity, and protection of pancreatic function. Evidence from human clinical trials remains limited, and the optimal quantity and variety of dates for safe consumption in diabetic patients have not been fully established. Variability in cultivar, ripening stage, and preparation methods may also affect glycemic outcomes, highlighting the need for more controlled studies to translate preclinical findings into clinical recommendations.

### 7.3 Anticancer bioactivities: preclinical evidence and translational limits

Globally, cancers account for one in six fatalities, presenting a serious health problem. Notwithstanding significant progress in medicine, cancer therapy continues to pose challenges. Plant-derived compounds are essential in the creation of new anticancer pharmaceuticals, offering researchers opportunities to identify and investigate novel chemicals with possible anticancer properties (Chunarkar-Patil et al., 2024).

Studies on the anticancer properties of dates are extremely scarce. Human evidence is very limited, primarily observational regarding colon health, while most data come from *in vitro* and animal models. Consuming dates may improve colon health by promoting the growth of beneficial gut bacteria, which can lead to reduced proliferation of tumor cells and a decreased risk of developing colon cancer (Eid et al., 2014).

*In vitro* studies indicate that date extracts exhibit moderate to high anticancer activity against various cancer cell lines. For example, the methanol extract of the date variant “Siwi” had the maximum preclinical anticancer activity with an IC50 of 99 ± 1.6 µg·mL−1, whereas the methanol extract of the date variant “Sukkari” showed an IC50 of 119 ± 3.5 µg·mL−1 against the human breast cancer cell line (MDA-MB-231) (Abdelbaky et al., 2023). Silver nanoparticles derived from Iraqi date palm also exhibited some degree of anticancer control against breast cancer cells (MCF7) compared to normal cells (MCF10A) (Al-Awady et al., 2019). Extracts of Jordanian date palm fruit demonstrated inhibitory effects on human mammary adenocarcinoma (MCF-7) cells *in vitro* (Khan et al., 2017).

Animal studies further support potential anticancer effects. The aqueous extract of Ajwa dates demonstrated anticancer effects in a diethylnitrosamine-induced hepatocellular carcinoma model using Wistar rats. Control contributed to the reversal of liver damage, restoring liver function, normalizing antioxidant enzymes, liver enzyme levels, cytokine balance, and gene expression, suggesting effective inhibition of hepatocellular carcinoma progression (Khan et al., 2017).

Additional *in vitro* findings include the Al-Zahdi date palm cultivar from Iraq, which exhibited anticancer activity with both oily and methanolic extracts against AMN3, HeLa, and Ref cancer cell lines (Al-Zeiny et al., 2022). The Medjool date cultivar also demonstrated significant anticancer activity against human breast adenocarcinoma MCF-7 cells (Rozila et al., 2018).

The above data indicate that dates contain bioactive compounds with anticancer potential, supported mainly by *in vitro* and limited animal studies. Observational evidence hints at benefits in colon health, and extracts from different cultivars show cytotoxic effects against several cancer cell lines. Human clinical evidence is extremely scarce. Most studies are limited to cell lines or animal models, making translation to clinical recommendations premature. Variability in cultivar, extraction methods, and assay conditions limits comparability across studies. Further controlled clinical studies are needed to confirm some anticancer features in humans.

### 7.4 Antimicrobial and antibiofilm properties: evidence and translational limits

With the increasing prevalence of multi-drug-resistant microorganisms, the current pace of antibiotic development is inadequate. As a result, plant-derived phytochemical compounds present a promising alternative, though innovative approaches are necessary to effectively harness these compounds and formulate them into viable drug therapies (Abdallah et al., 2023). Research on date fruits has revealed notable antimicrobial activities. For example, the Sokkari date variety, cultivated in Saudi Arabia, was evaluated against six fungal strains, with Fusarium graminearum and Penicillium sp. exhibiting high sensitivity. Significant antibacterial activity was also observed against all tested bacterial strains, including Bacillus subtilis, Escherichia coli, and Pseudomonas aeruginosa (Ismail and Altuwairki, 2016). Methanolic extracts from five Tunisian date varieties (Beidh Hmam, Degla, Khalt Ahmar, Rtob, Rtob Hodh) demonstrated varying levels of antibacterial activity against pathogenic bacteria, with stronger effects observed against Gram-positive strains (Vayalil, 2012). Similarly, water and ethanol extracts of an Egyptian date fruit exhibited significant antibacterial activity against Salmonella enterica (20 ± 0.54 mm and 14 ± 0.52 mm), E. coli (20 ± 0.57 mm and 16 ± 0.57 mm), and Bacillus subtilis (18 ± 0.32 mm and 15 ± 0.23 mm), along with moderate inhibition of Staphylococcus aureus (8 ± 0.48 mm and 5 ± 0.52 mm) and Enterococcus faecalis (5 ± 0.36 mm and 2 ± 0.57 mm) (El Sohaimy et al., 2015). Phenolic compounds from several Saudi date cultivars (Ajwa, Safawi, Khalas, and Sukkary) also showed significant antimicrobial activity, with the Ajwa cultivar exhibiting the highest inhibition, particularly against Staphylococcus aureus, Salmonella enteritidis, and Candida albicans, as well as notable suppression of biofilm formation (reductions of 70.5% and 54.19% against Staphylococcus and Salmonella, respectively (Al-Tamimi et al., 2021). Eco-friendly silver nanoparticles derived from Iraqi date palm varieties demonstrated potent antibacterial activity against methicillin-resistant Staphylococcus aureus (MRSA) (Al-Awady et al., 2019). In addition, extracts from the fresh fruit of the Hayany variety showed strong activity against multi-drug-resistant strains of Candida albicans and Staphylococcus aureus, and their combination with amikacin further enhanced antimicrobial efficacy (Halabi et al., 2022).

*In vitro* studies consistently demonstrate strong antimicrobial potential of date fruit extracts across multiple bacterial and fungal strains, though no animal or human clinical studies are currently available. The activity varies depending on cultivar, extraction method, and solvent used. The current evidence is primarily from *in vitro* studies, which limits direct extrapolation to humans. Standardization of cultivar, extract preparation, and dosage is lacking, and mechanistic insights into the antimicrobial action remain limited. Synergistic effects with antibiotics are promising but require further validation.

Date fruit extracts, especially from Ajwa, Sokkari, and Hayany cultivars, exhibit strong *in vitro* antimicrobial activity. Future research should focus on *in vivo* animal studies and controlled human trials to determine efficacy, safety, and optimal dosage, enabling potential therapeutic applications against microbial infections.

### 7.5 Antiviral bioactivities: evidence and research gaps

Viruses are serious infectious agents that are challenging to treat and have caused millions of fatalities worldwide throughout human history. Treatment options remain limited, with few effective drugs and vaccines available (Kausar et al., 2021). The need for new antiviral agents has grown significantly, especially following the COVID-19 pandemic, which highlighted the challenges healthcare professionals face in controlling viral outbreaks even with the availability of vaccines. Various medicinal plants have been documented for their antiviral potential, and certain edible fruits such as citrus, almonds, apples, blackberries, grapes, black currants, cranberries, Japanese cherry, pomegranate, mango, mulberry, pistachios, and strawberries have demonstrated promising antiviral properties against a range of pathogenic viruses (Santhi et al., 2021). Studies on the antiviral activities of date fruits, however, remain scarce. *In vitro* research evaluated the antiviral properties of acetone date pit extract against Pseudomonas phage ATCC 14209-B1, using Pseudomonas aeruginosa as the host cell, and reported a significant reduction in phage infectivity (Jassim and Naji, 2010). In silico studies also indicated that compounds such as apigenin, luteolin, and diosmetin from date fruits possess potential antiviral activity, including anti-SARS-CoV-2 effects (Arifin and Febriansah, 2022).

The current evidence for antiviral activity of dates is primarily limited to *in vitro* and in silico studies, showing potential but not yet validated *in vivo* or in clinical settings. The antiviral effect appears to be associated with specific phytochemicals such as flavonoids. There is a lack of animal or human studies evaluating antiviral efficacy. The mechanisms of action remain poorly understood, and standardization of extracts or active compounds has not been established.

Date fruits show preliminary antiviral potential, particularly due to compounds like apigenin, luteolin, and diosmetin. Future research should focus on well-designed *in vivo* and clinical studies to confirm these effects, elucidate mechanisms, and identify safe and effective dosages for antiviral applications.

### 7.6 Date palm fruits and general well-being: evidence and limitations

Human well-being is linked to good health and is a comprehensive concept encompassing physical, mental, emotional, social, and spiritual health, all of which contribute to a person’s overall quality of life (Friedman and Kern, 2014). Numerous studies have demonstrated the capacity of palm dates to provide a range of beneficial activities, including antioxidant, antimutagenic, anticancer, antibacterial, anti-inflammatory, gastroprotective, hepatoprotective, nephroprotective, antihyperlipidemic, antiproliferative, and immunostimulant effects (Baliga et al., 2011; Abdul-Hamid et al., 2020; Ahmad Mohd Zain et al., 2022). Notably, anti-inflammatory properties of dates have been observed in a rat adjuvant arthritis model. Additionally, consumption of dates has been shown to enhance plasma antioxidant levels, including vitamins C, E, A, and β-carotene, while reducing lipid peroxide levels, thereby contributing to overall health promotion (Baliga et al., 2011). Beyond being rich in essential minerals, vitamins, and sugars for energy (Al-Farsi and Lee, 2008), dates have also been reported to enhance sexual function in both men and women (Mirza et al., 2019). Date palm kernel extracts exhibit anti-aging properties, suggesting their potential application in skincare products to mitigate aging effects. Date palm seed oil has been utilized in the formulation of creams, liquid shampoos, and bar shaving soaps, while natural wax extracted from date palm leaflets offers potential for cosmetics and healthcare applications (EL-Mously et al., 2023). Regular consumption of dates may provide hypothesis-generating effects in disease prevention through modulation of anti-inflammatory, antioxidant, and anti-tumor activities (Rahmani et al., 2014).

The benefits of dates on general health and well-being are supported by both *in vivo* animal studies and limited human observations. Evidence shows that dates can enhance antioxidant status, reduce inflammation, and contribute to metabolic and reproductive health. Many functional applications, such as anti-aging and cosmetic uses, are supported by experimental data on extracts and oils. Most studies are preclinical or observational, and human clinical trials are limited. Standardization of active compounds and dosage for specific health outcomes has not been established. Evidence for claims such as sexual function improvement or cosmetic benefits remains preliminary and requires further validation in controlled human studies.

Date fruits offer multiple potential benefits for overall health and well-being, including antioxidant, anti-inflammatory, metabolic, and cosmetic effects. Future human clinical trials and standardized intervention studies are needed to confirm these effects and provide evidence-based guidelines for daily consumption.

## 8 Future perspectives

Although research on the nutritional composition and biological activities of *Phoenix dactylifera* L. fruits has expanded, important gaps remain. A key priority is the standardization of reporting and experimental conditions, as variation in cultivar, ripening stage, geographic origin, and postharvest processing limits cross-study comparability. Harmonized criteria for describing these variables would improve data consistency and interpretation (Alu’datt et al., 2025). Future studies should also apply advanced chemical profiling approaches, including metabolomics and comprehensive chromatographic analyses, to better characterize the complex phytochemical composition of date fruits and to identify compound patterns associated with specific biological effects, rather than relying on individual markers (Sejpal et al., 2025). In parallel, greater attention to digestive and metabolic processes is needed to clarify how date-derived bioactive compounds are absorbed, transformed, and utilized following consumption.

Artificial intelligence offers new opportunities to enhance food science, quality assessment, and safety evaluation through data-driven modeling and image analysis, which can be applied for dates production. However, effective application requires standardized datasets, regulatory alignment, and scalable frameworks adapted to diverse date-producing regions (Mir et al., 2026). In addition, exploring broader food-system applications, such as the use of dates or their components in functional and formulated foods, may expand their nutritional relevance beyond traditional consumption patterns (Alu’datt et al., 2025). Finally, most reported biological activities of date palm fruits are based on *in vitro* studies, whereas *in vivo* investigations remain limited. This imbalance highlights a clear research gap that must be addressed in the future studies to rigorously validate proposed biological properties and to assess their relevance under physiologically meaningful conditions.

## 9 Conclusion

Date palm (*Phoenix dactylifera* L.) fruits are an important food in many regions and provide carbohydrates, dietary fiber, minerals, and diverse phytochemicals such as phenolic acids, flavonoids, carotenoids, and phytosterols. These components explain why dates are often linked to antioxidant and metabolism-related biological activities. However, most reported biological effects of date palm fruits are based on *in vitro* experiments, while *in vivo* and human studies remain limited. In addition, large differences in cultivar, ripening stage, processing methods, and experimental design make it difficult to compare results between studies. For these reasons, the practical relevance of many claimed health benefits is still uncertain. Future research should move beyond descriptive studies and focus on standardized methods, realistic intake levels, and biologically meaningful outcomes. Greater attention to cultivar-specific properties and clearer reporting of experimental conditions will improve reproducibility. Collaboration between plant scientists, food researchers, and nutrition scientists will also be important. In summary, date palm fruits should be considered as a functional food as it has clear nutritional value and promising bioactive potential beside its Contribution to gut health, energy balance, and micronutrient intake. Turning this potential into reliable and practical applications will require stronger evidence, better study design, and closer alignment between experimental findings and real dietary conditions.
