# Supplementary material for: Date palm fruits (Phoenix dactylifera L.): nutrition, phytochemistry, and translational gaps in reported bioactivities — a critical narrative review
Source: Front Plant Sci. 2026 Apr 13;17:1806447. doi: 10.3389/fpls.2026.1806447 (PMC13111235; doi:10.3389/fpls.2026.1806447)
Supplement: Supplementary file 1 [file DataSheet1.docx]

Supplementary Material

**Table S1.** Total sugar composition (g /100 g dw) in different Palm varieties at the mature stage

| **Reference** | **Date palm variety** | **Total sugars (g/100 g dw)** |
| --- | --- | --- |
| **Aljaloud et al. (2020)** | Ajwa | 74.3 |
|  | Majdoul | 66.47 |
|  | Deglet Noor | 86.42 |
| **Assirey (2015)** | Ajwa | 74.3 |
|  | Shalaby | 75.9 |
|  | Khodari | 79.4 |
|  | Anabarah | 78.4 |
|  | Sukkari | 78.5 |
|  | Suqaey | 79.7 |
|  | Safawy | 75.3 |
|  | Buri | 81.4 |
|  | Labanah | 71.2 |
|  | Mabroom | 76.4 |
| **Al-Shahib & Marshall (2003)** | Barhi | 57.2 |
|  | Buchibal | 55.1 |
|  | Fard | 59.5 |
|  | Gush Rabei | 49.9 |
|  | Hilali Ahmr | 64.1 |
|  | Hilal Pakistan | 51.4 |
|  | Khasab | 60.6 |
|  | Khulas | 57.0 |
|  | Khumaizy | 53.9 |
|  | Lulu | 57.7 |
|  | Naghal | 44.3 |
|  | Naghal Hilali | 52.7 |
| **Al-Farsi & Lee (2008)** | Naghal | 52.6 |
|  | Khunaizy | 58.8 |
|  | Khalas | 56.8 |
|  | Barhi | 56.9 |
|  | Lulu | 52.2 |
|  | Fard | 59.4 |
|  | Khasab | 47.8 |
|  | Bushibal | 54.2 |
|  | Gash Gaafar | 57.0 |

**Table S2.** Sugar contents of different date fruit varieties according to many authors.

| **Glucose** | **Fructose** | **Sucrose** | **Maltose** | **Galactose** | **Reference** |
| --- | --- | --- | --- | --- | --- |
| 31.3 – 47.4 | 32.1 – 39.3 | 4.2 – 6.9 | – | – | Aljaloud et al. (2020) |
| 35.4 – 54.5 | 39.4 – 52.5 | 0.0 – 13.4 | 22.5 | 12.2 | Khalid et al. (2017) |
| 37.3 – 52.3 | 28.1 – 48.3 | 2.9 – 4.9 | 22.5 | 12.2 | Assirey (2015) |
| 1.5 – 95.4 | 53 – 113 | 9.23 – 139 | – | – | Hamad et al. (2015) |
| 29.04 – 34.53 | 20.72 – 23.65 | 1.86 – 2.34 | – | – | Al-Shahib and Marshall (2003) |

**Table S3.** Protein content in different cultivar of date palm fruit

| **Number of cultivars** | **Protein concentration (g/100 g dw)** | **Reference** |
| --- | --- | --- |
| 9 | 1.72 – 4.73 | Assirey (2015) |
| 14 | 2.63 – 3.78 | Al-Harrasi et al. (2014) |
| 9 | 1.60 – 2.50 | Borchani et al. (2010) |

**Table S4.** Amino acid concentration of date palm fruit reported from different studies.

| **Amino acids** | **Assirey (2015)** | **Auda et al. (1976)** | **Ahmed et al. (1995); Ahmed et al (2013) Al-Farsi et al. (2005)** |
| --- | --- | --- | --- |
| Alanine (Ala) | 78 – 105 | 30 – 68 | 8 – 342 |
| Arginine (Arg) | 37 – 93 | 34 – 76 | 2 – 261 |
| Aspartic + Asparagine | 127 – 225 | 72 – 183 | 230 – 450 |
| Cysteine (Cys) | 32 – 46 | 13 – 48 | 11 – 114 |
| Glutamic + Glutamine | 158 – 265 | 100 – 217 | 105 – 718 |
| Glycine (Gly) | 83 – 102 | 42 – 86 | 4 – 349 |
| Histidine (His) | 21 – 27 | 14 – 34 | 0.1 – 76 |
| Isoleucine (Ile) | 43 – 55 | 9 – 34 | 0.2 – 465 |
| Leucine (Leu) | 57 – 100 | 41 – 94 | 0.5 – 264 |
| Lysine (Lys) | 51 – 73 | 53 – 136 | 3 – 282 |
| Methionine (Met) | 17 – 27 | 4 – 13 | 0.2 – 219 |
| Phenylalanine (Phe) | 45 – 56 | 25 – 51 | 0.8 – 219 |
| Proline (Pro) | 86 – 113 | 36 – 73 | 12 – 369 |
| Serine (Ser) | 57 – 64 | 29 – 70 | 6 – 238 |
| Threonine (Thr) | 42 – 53 | 23 – 57 | 1 – 264 |
| Tryptophan (Trp) | 13 – 46 | – | 100 |
| Tyrosine (Tyr) | 15 – 40 | 16 – 34 | 1 – 181 |
| Valine (Val) | 65 – 86 | – | 0.5 – 271 |

**Table S5.** Fatty acid composition of date seeds provided from some studies

| **C8:0** | **C10:0** | **C12:0** | **C14:0** | **C16:0** | **C17:0** | **C18:0** | **C20:0** | **C21:0** | **C22:0** | **C23:0** | **C16:1** | **C18:1** | **C18:2** | **C18:3** | **Reference** |
| --- | --- | --- | --- | --- | --- | --- | --- | --- | --- | --- | --- | --- | --- | --- | --- |
| 0.0–0.8 | 0.0–0.6 | 8.4–24.1 | 10.6–14.5 | 11.1–13.0 | – | 2.7–5.5 | – | – | – | – | – | 40.6–52.8 | 6.0–10.1 | – | Al-Shahib and Marshall, (2003) |
| 6.3–7.1 | 5.2–10.9 | 5.3–13.8 | 10.6–12.0 | 1.4–3.7 | 0.7–3.0 | 0.5–0.8 | 0.6–0.7 | – | – | – | – | 57.1–58.3 | 11.6–58.8 | 0.1–12.8 | Al-Hooti et al., 1997 |
| 0.3–0.5 | 15.4–24.7 | 7.4–11.8 | 6.7–10.1 | 0.1–0.5 | 0.2–1.3 | 0.5–1.3 | 0.1–0.6 | 0.2–2.2 | 0.1 | 0.1–0.5 | – | 42.6–56.9 | 0.2–3.4 | 0.3–1.3 | Al-Showiman et al., 1998 |

**Table S6.** Macro and microelement concentrations of different varieties of dates at different ripening stages (10^-2^g/kg^-1^). (Al-Hooti et al., 1997; Rastegar et al., 2012).

| **Mineral** | **K** | **Ca** | **Mg** | **Na** | **Mn** | **Zn** | **Fe** |
| --- | --- | --- | --- | --- | --- | --- | --- |
| **Concentration** | 2720 – 1170 | 490 – 140 | 370 – 114 | 133 – 120 | 3.6 – 0.4 | 3.5 – 1.4 | 3.5 – 1.2 |

**Table S7.** Macro and microelement concentrations of different varieties of dates at different ripening stages (mg/100g dw). (Al-Hooti et al., 1997; Rastegar et al., 2012).

| **Mineral** | **K** | **Ca** | **Mg** | **Na** | **Zn** | **Fe** |
| --- | --- | --- | --- | --- | --- | --- |
| **Concentration** | 752.6 – 107.4 | 142.4 – 36.3 | 132.7 – 46.1 | 9.7 – 2.5 | 1.6 – 0.3 | 8.1 – 0.9 |

**References**

Ahmed, I. A., Ahmed, A. W. K., and Robinson, R. K. (1995). Chemical composition of date varieties as influenced by the stage of ripening. *Food Chem.* 54, 305–309.

Ahmed, J., Al-Jasass, F. M., and Siddiq, M. (2013). Date fruit composition and nutrition. In: *Dates: Postharvest Science, Processing Technology and Health Benefits*, 261–283.

Al-Farsi, M., Alasalvar, C., Morris, A., Baron, M., and Shahidi, F. (2005). Compositional and sensory characteristics of three native sun-dried date (*Phoenix dactylifera* L.) varieties grown in Oman. *J. Agric. Food Chem.* 53, 7586–7591.

Al-Farsi, M. A., and Lee, C. Y. (2008). Nutritional and functional properties of dates: a review. *Crit. Rev. Food Sci. Nutr.* 48, 877–887.

Al-Harrasi, A., Rehman, N. U., Hussain, J., Khan, A. L., Al-Rawahi, A., Gilani, S. A., et al. (2014). Nutritional assessment and antioxidant analysis of 22 date palm (*Phoenix dactylifera*) varieties growing in Sultanate of Oman. *Asian Pac. J. Trop. Med.* 7, S591–S598.

Al-Hooti, S., Sidhu, J., and Qabazard, H. (1997). Physicochemical characteristics of five date fruit cultivars grown in the United Arab Emirates. *Plant Foods Hum. Nutr.* 50, 101–113.

Aljaloud, S., Colleran, H. L., and Ibrahim, S. A. (2020). Nutritional value of date fruits and potential use in nutritional bars for athletes. *Food Nutr. Sci.* 11, 463–480.

Al-Shahib, W., and Marshall, R. J. (2003). The fruit of the date palm: its possible use as the best food for the future? *Int. J. Food Sci. Nutr.* 54, 247–259.

Al-Showiman, S. (1998). *Al Tamr, Ghetha wa Saha (Date, Food and Health).* Saudi Arabia: Dar Al-Khareji Press.

Assirey, E. A. R. (2015). Nutritional composition of fruit of 10 date palm (*Phoenix dactylifera* L.) cultivars grown in Saudi Arabia. *J. Taibah Univ. Sci.* 9, 75–79.

Auda, H., Al-Wandawi, H., and Al-Adhami, L. (1976). Protein and amino acid composition of three varieties of Iraqi dates at different stages of development. *J. Agric. Food Chem.* 24, 365–367.

Borchani, C., Besbes, S., Blecker, C., Masmoudi, M., Baati, R., and Attia, H. (2010). Chemical properties of 11 date cultivars and their corresponding fiber extracts. *Afr. J. Biotechnol.* 9, 4096–4105.

Hamad, I., AbdElgawad, H., Al Jaouni, S., Zinta, G., Asard, H., Hassan, S., et al. (2015). Metabolic analysis of various date palm fruit (*Phoenix dactylifera* L.) cultivars from Saudi Arabia to assess their nutritional quality. *Molecules* 20, 13620–13641.

Khalid, S., Khalid, N., Khan, R. S., Ahmed, H., and Ahmad, A. (2017). A review on chemistry and pharmacology of Ajwa date fruit and pit. *Trends Food Sci. Technol.* 63, 60–69.

Rastegar, S., Rahemi, M., Baghizadeh, A., and Gholami, M. (2012). Enzyme activity and biochemical changes of three date palm cultivars with different softening pattern during ripening. *Food Chem.* 134, 1279–1286.
